# Impact of Surfactant Protein-A Variants on Survival in Aged Mice in Response to *Klebsiella pneumoniae* Infection and Ozone: Serendipity in Action

**DOI:** 10.3390/microorganisms8091276

**Published:** 2020-08-21

**Authors:** Nithyananda Thorenoor, David S. Phelps, Padma Kala, Radhika Ravi, Andreas Floros Phelps, Todd M. Umstead, Xuesheng Zhang, Joanna Floros

**Affiliations:** 1 Center for Host Defense, Inflammation, and Lung Disease (CHILD) Research, Department of Pediatrics, The Pennsylvania State University College of Medicine, Hershey, PA 17033, USA; dphelps@pennstatehealth.psu.edu (D.S.P.); tumstead2@pennstatehealth.psu.edu (T.M.U.); hmczxs@gmail.com (X.Z.); 2 Department of Biochemistry and Molecular Biology, The Pennsylvania State University College of Medicine, Hershey, PA 17033, USA; 3 Independent Consultant, Upper Saddle River, NJ 07458, USA; pkala_19044@yahoo.com; 4 Division of Anesthesia, Department of Surgery, Veterans Affairs New Jersey Health Care System, 385 Tremont Avenue, East Orange, NJ 07018, USA; radhika.ravi@gmail.com; 5 The Collective Potential, San Francisco, CA 94117, USA; andreas@thecollectivepotential.com; 6 Department of Obstetrics & Gynecology, The Pennsylvania State University College of Medicine, Hershey, PA 17033, USA

**Keywords:** pneumonia infection, surfactant protein A1 and A2, filtered air (FA), ozone (O_3_)

## Abstract

Innate immune molecules, SP-A1 (6A^2^, 6A^4^) and SP-A2 (1A^0^, 1A^3^), differentially affect young mouse survival after infection. Here, we investigated the impact of SP-A variants on the survival of aged mice. hTG mice carried a different SP-A1 or SP-A2 variant and SP-A-KO were either infected with *Klebsiella pneumoniae* or exposed to filtered air (FA) or ozone (O_3_) prior to infection, and their survival monitored over 14 days. In response to infection alone, no gene- or sex-specific (except for 6A^2^) differences were observed; variant-specific survival was observed (1A^0^ > 6A^4^). In response to O_3_, gene-, sex-, and variant-specific survival was observed with SP-A2 variants showing better survival in males than females, and 1A^0^ females > 1A^3^ females. A serendipitous, and perhaps clinically important observation was made; mice exposed to FA prior to infection exhibited significantly better survival than infected alone mice. 1A^0^ provided an overall better survival in males and/or females indicating a differential role for SP-A genetics. Improved ventilation, as provided by FA, resulted in a survival of significant magnitude in aged mice and perhaps to a lesser extent in young mice. This may have clinical application especially within the context of the current pandemic.

## 1. Introduction

*Klebsiella pneumoniae* (*K. pneumoniae*), a Gram-negative bacterium, was first isolated from patients with pneumonia [1] and is found in nature including water, soil, and animals, and it can colonize medical devices and health care environments [2,3,4,5]. It is an opportunistic pathogen colonizing mucosal surfaces including the gastrointestinal tract and oropharynx [4,5,6], without causing pathology and may disseminate to other tissues causing life-threatening infection including pneumonia, urinary-tract infections (UTIs), bloodstream infection, and sepsis [7]. *K. pneumoniae* infection is a major health problem in neonates, the elderly, and immunocompromised individuals within the healthcare setting [8], and is responsible for community-acquired infections worldwide [9]. A significant feature of the *K. pneumoniae* infection is its ability to metastatically spread and significantly increase mortality and morbidity [7].

Collectins are a family of proteins that contains the mannose-binding protein and lung surfactant proteins (SPs), SP-A and D [10]. SP-A is a major surfactant host defense molecule involved in innate immunity [11,12,13]. It consists of an N-terminal region, a collagen-like domain, a neck, and a C-terminal carbohydrate recognition domain that recognizes and binds specific glycoproteins, debris, pathogens, and allergens [13,14,15,16]. SP-A is involved in multiple alveolar macrophage (AM)-mediated host defense functions such as the stimulation of chemotaxis of macrophages [17], enhancement of phagocytosis of bacteria by macrophages [18,19], proliferation of immune cells [20,21], and linkage of innate and adaptive immunity [22]. Several studies have shown that susceptibility to pneumonia and other types of lung injury are increased in the absence of SP-A [23,24,25,26,27].

Human SP-A consists of two functional genes, *SFTPA1* and *SFTPA2*, encoding SP-A1 and SP-A2 proteins, respectively, and each has been identified with a number of variants [28,29]. SP-A gene variants that may have an impact on function are classified based on their nucleotide differences within the coding sequences [28,29,30,31]. We and others have demonstrated differences between SP-A1 and SP-A2 variants both qualitative (i.e., functional, biochemical, and/or structure) [32,33,34,35,36,37,38,39,40,41,42,43,44,45] and quantitative (regulatory) [46,47,48,49,50,51,52,53,54,55,56]. Moreover, SP-A1 and SP-A2 variants differ in their ability to modulate gene expression and the proteomic expression profile of AM and the AM actin cytoskeleton [57,58,59,60]. A single-cell analysis based on actin-staining revealed alveolar macrophage phenotypic subpopulation as well as sex- and age-related differences in KO mice in response to SP-A1 and SP-A2 proteins [59]. Sex differences in survival and lung function mechanics in response to bacterial infection between SP-A1 and SP-A2 and among variants have been observed [39,40]. Furthermore, the SP-A variant-dependent AM gene expression in response to infection varies in a sex-specific manner [60]. SP-A1 compared to SP-A2 exhibits a higher efficiency in pulmonary surfactant reorganization and surfactant inhibition by serum proteins [61], whereas SP-A2 exhibits higher activity in host defense-related functions [35,39,45]. The major contributor for at least some of these differences appears to be amino acid 85 of the precursor molecule, where SP-A1 has a cysteine and SP-A2 has an arginine [31,42].

Studies in humans and animal models have shown that macrophage function becomes compromised with advancing age, and this age-dependent dysfunction may include chemotaxis, phagocytosis, production of reactive oxygen species, and regulation of cytokine responses [62,63]. Differences between young and old, in terms of a reduced production of TNF-α, and IL-10 with aging [64,65] and an impaired production of pro- and anti-inflammatory cytokines, may collectively increase the risk for infection and inability to resolve inflammation [63]. Ozone (O_3_) is a major constituent of air pollution formed by the photochemical reactions of carbon monoxide, nitrogen oxides, and chemically-active hydrocarbons [66,67]. Short-term exposure to O_3_ is associated with an increased incidence of respiratory ailments [68,69,70,71], and females are at increased risk of adverse health outcomes from O_3_ than males [72,73,74,75,76]. Human and animal model studies have shown that short-term exposure to O_3_ was significantly associated with increased risk of mortality in old age [77,78]. Previously, we have studied the survival of young *K. pneumoniae*-infected mice in response to different SP-A1 and SP-A2 variants [39], as well as in the absence of SP-A [24]. In the current study, we investigated whether the genetics of innate immunity, and especially those of SP-A1 and SP-A2, differentially affect the survival of aged male and female mice in response to infection, as observed in young mice, and in response to O_3_ or filtered air exposure prior to infection.

In the present study, building on our previous findings, we investigated the role of two SP-A1 (6A^2^, 6A^4^) and two SP-A2 (1A^0^, 1A^3^) variants, which are frequently observed in the general population [28], on survival, in response to *K. pneumoniae* alone and in response to filtered air (FA) or ozone (O_3_)-exposure prior to infection in aged mice (~9–12 months). We observed in response to infection alone, no gene- or sex-specific (except for SP-A1 (6A^2^)) differences in survival, but variant-specific survival was observed. In mice that received either FA or O_3_ exposure prior to infection, we observed gene-, sex-, and variant-specific differences in survival rates. O_3_-exposed SP-A2 (1A^0^) males showed a similar survival to that of FA-exposed mice as well as a better survival than 1A^0^ females. Surprisingly and unexpectedly, the survival of mice exposed to FA (used as a control for O_3_ exposure) prior to infection exhibited significantly better survival than infected mice alone (no FA exposure). These observations indicate that the genetics of the innate immune molecule SP-A have a) a differential impact on survival in aged mice, under different conditions, and b) increased ventilation (i.e., high flow rate of FA) has an absolutely positive impact on survival of a significant magnitude in aged mice and to lesser degree on young mice as shown in a pilot study. These observations may be directly relevant in the clinical setting.

## 2. Materials and Methods

### 2.1. Animals

Humanized transgenic (hTG) mice that each carried SP-A1 (6A^2^, 6A^4^), SP-A2 (1A^0^, 1A^3^), as well as SP-A-knockout (KO) mice were used in the present study. hTG mice were generated on the C57BL6/J SP-A-KO background as described previously [79]. All mice were ~9–12 months old, and were maintained as described [40]. Both males and females were studied. The females were synchronized with regard to estrous cycle as described [40]. A total of 643 mice were used. Of these 31 mice, were young mice (3–4 months). The Penn State Hershey Medical Center Institutional Animal Care and Use Committee (IACUC) approved all procedures 44968 involving animals.

### 2.2. Preparation of Bacteria

*K. pneumoniae* bacteria (ATCC 43816) were obtained from American Tissue Culture Collection (Rockville, MD) and prepared as described previously [24,39,40,80]. In brief, bacteria were grown at 37 °C for 18 h in Tryptic soy broth (TSB, Sigma-Aldrich, St. Louis, MO, USA) media to reach stationary phase. The overnight bacterial cultures were used to inoculate a sub-culture in 50 mL of fresh TSB for 3 h to reach the mid-log phase of growth. The growth was stopped by keeping the subculture on ice and serially diluted in PBS (Corning, NY, USA) to obtain ~3.6 × 10^4^ CFU/mL. Fifty µl of a bacterial suspension containing ~ 1800 CFU was used to infect each mouse. CFU per ml values were estimated based on the standard curve obtained at OD_660_ of the bacterial suspension.

### 2.3. Infection of Mice with K. Pneumoniae

Infection for the young mice was performed as described previously [24] and for the aged mice as described previously [24], except a significantly higher CFU (~1800) inoculum was used for the aged mice. The higher dose was needed, because unlike the younger mice, all of the aged mice recovered if they were infected with the low CFU (~450) inoculum used for the young mice [39,40,60]. Briefly, hTG mice, SP-A1 (6A^2^, 6A^4^), SP-A2 (1A^0^, 1A^3^), and SP-A-KO male and female mice were anesthetized with a mixture of ketamine (Vedco. Inc, St. Joseph, MO, USA) and xylazine (Akorn. Inc, lake forest, IL, USA) and infected with *K. pneumoniae* (~1800 CFU/mouse) in 50 µL of PBS delivered to oropharynx and aspirated after a brief nasal occlusion [60,81]. The mice were monitored for survival twice a day (morning and evening, 6–8 h difference between the two daily observations and at each observation time point, each mouse was observed for 5–10 min) for 14 days after infection. If the infected mice were obviously sick (unkempt, hunched, isolated from cage mates, eyelids are more tightly closed than normal, not moving when disturbed, unsteady gait, immobile, unable to remain upright, and unresponsive to external stimuli) and considered to have no chance of recovery, they were euthanized immediately to prevent further suffering according to Penn State University IACUC protocol.

### 2.4. Filtered Air (FA) and Ozone (O_3_) Exposure and Infection

The animals were exposed to FA or O_3_ in parallel prior to infection as described previously [23,82]. The air is from a centralized-air source and for both FA and O_3_ systems is conveyed through a 5-µm particle filter, 0.5-µm and 0.1-µm coalescing filters, a carbon filter, and a membrane drier and with an airflow of 15 L/min [82] that is subsequently warmed and humidified prior to delivery to the exposure vessels. In the case of the O_3_ system, the O_3_ was generated from oxygen by an electrical discharge ozonizer and added to the conditioned filtered air. The oxygen flow to the ozonizer was controlled by two mass flow units, one was used to exhaust excess ozone in oxygen at a flow rate of 725 mL/min and the other was used to introduce ozone to the conditioned filtered air at a flow rate of about 25 mL/min. The air flow through both chambers (FA and O_3_) was adjusted to be identical [82]. Male and female mice were exposed to FA or FA containing 2 ppm O_3_ for 3 h. The mice were anesthetized and infected with *K. pneumoniae* as described above immediately after the exposure.

### 2.5. Statistical Analysis

Survival was analyzed by log-rank test (cumulative survival, for the entire 14 days period), and with a Chi-Square test (daily survival). The surviving animals were compared with a one-way analysis of variance (ANOVA) followed by Bonferroni multiple comparisons correction for each experimental group. Data are presented as mean with ± standard deviation. *p*-value < 0.05 was considered to be significant (GraphPad Prism version 5; GraphPad Software, San Diego, CA, USA).

## 3. Results

Two general groups of hTG mice, SP-A1 (6A^2^, 6A^4^), SP-A2 (1A^0^, 1A^3^), and SP-A-KO male and female mice (~9–12 months aged) were studied: a) one group was infected with *K. pneumoniae*, and b) the other group was exposed to filtered air (FA) or ozone (O_3_) before infection. The mice of both groups were monitored for 14 days for their survival. A total of 612 mice were used.

### 3.1. Effect of SP-A Variants on Survival after Infection

**Figure 1:**
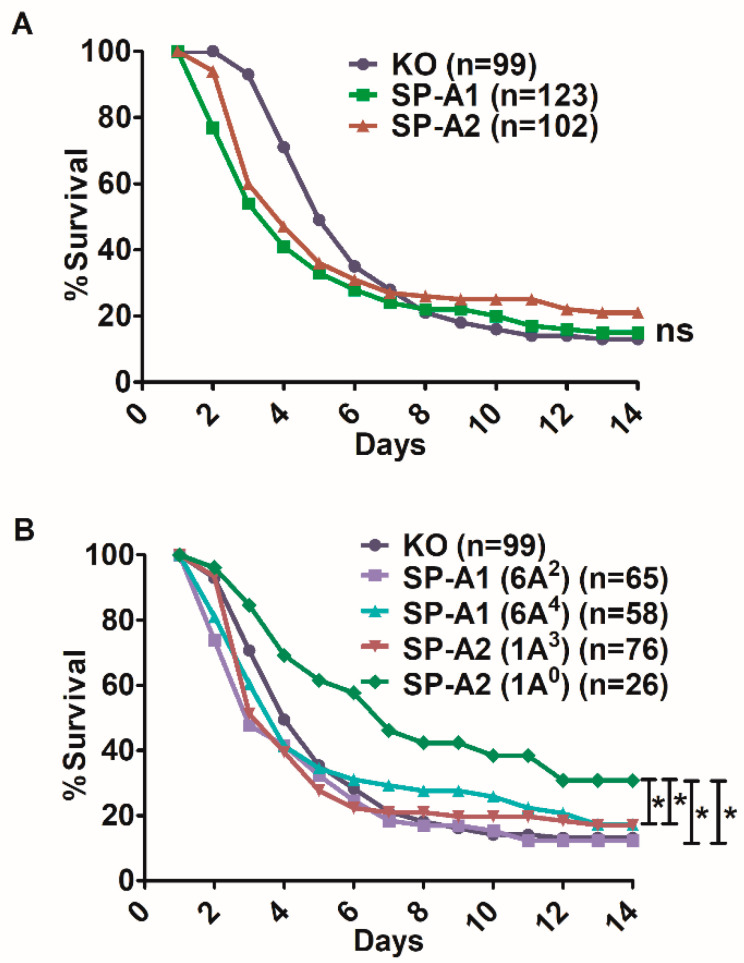
Comparison of survival after *K. pneumoniae* infection. Panel (**A**) depicts the daily survival curves of the combined male and female mice carrying a single SP-A1 or SP-A2 variant and of mice lacking SP-A (KO). Panel (**B**) depicts the survival curves of the combined male and female mice of KO, for each SP-A2 (1A^0^, 1A^3^), and SP-A1 (6A^2^, 6A^4^) variant. Significant differences are indicated for survival * *p* < 0.05 (log-rank test and one-way analysis of variance (ANOVA) followed by Bonferroni multiple comparisons correction), ns: not significant.

When we compared the overall survival of mice carrying SP-A1 variants (6A^2^, 6A^4^) and SP-A2 variants (1A^0^, 1A^3^) we found that all mice had a similar survival compared to KO indicating that the presence or the absence of SP-A did not alter the outcome in aged mice after *K. pneumoniae* infection when the males and females were combined and the variants of each gene were also combined (Figure 1A).

Next, we investigated whether survival differences exist among variants (Figure 1B). Mice carrying an SP-A2 (1A^0^) single gene variant had significantly higher survival than SP-A1 (6A^2^, 6A^4^), SP-A2 (1A^3^), and KO. The SP-A1 (6A^2^, 6A^4^) or SP-A2 (1A^3^) variants had a similar survival rate with the KO (Figure 1B).

### 3.2. Sex Differences in Survival of SP-A1, SP-A2, and KO Mice

**Figure 2:**
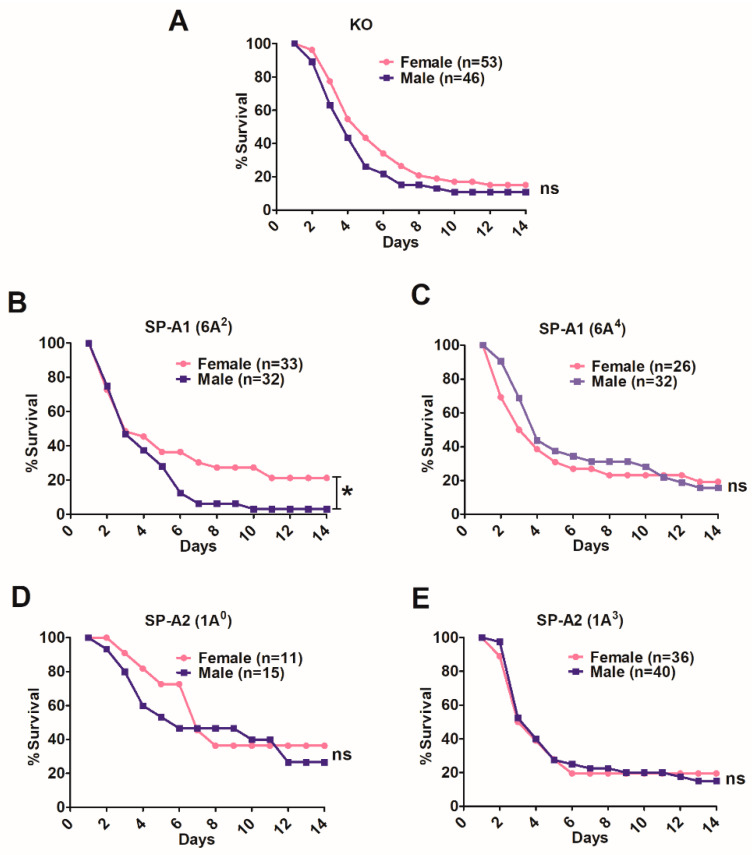
Effect of sex on survival after *K. pneumoniae* infection. The survival rate was measured in SP-A-KO (panel (**A**)), SP-A1 (6A^2^, 6A^4^) (panels (**B**,**C**)), and SP-A2 (1A^0^, 1A^3^) (panels (**D**,**E**)) male and female mice over a period of 14 days. Significant differences for survival are indicated * *p* < 0.05 (Chi-Square and log-rank test). The number of mice used in each group is shown in Figure panels in parenthesis (*n* =), ns: not significant.

In the SP-A1 (6A^4^), SP-A2 (1A^0^, 1A^3^), and KO groups no significant sex differences in survival were observed over the 14-day observation period (Figure 2A,C–E). However, the SP-A1 (6A^2^) exhibited significant sex differences. Males compared to females showed a significant decrease in survival (Figure 2B).

#### 3.2.1. Differences between Gene-Specific Variants

**Figure 3:**
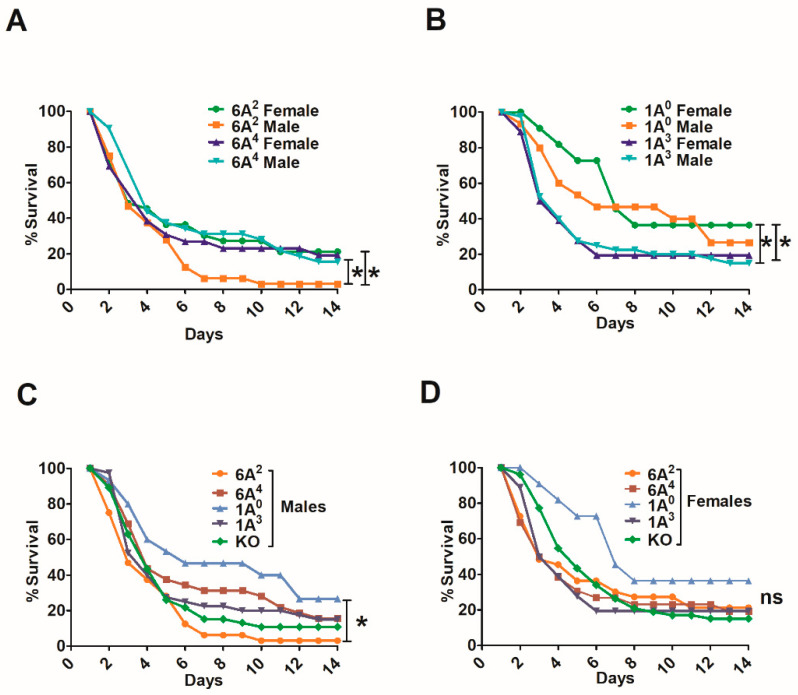
Survival as a function of SP-A gene-specific variant and sex after *K. pneumoniae* infection. Panel (**A**) depicts differences in survival between male and female mice of SP-A1 (6A^2^, 6A^4^) and Panel (**B**) depicts differences of SP-A2 (1A^0^, 1A^3^) variants. Panel (**C**) depicts differences in survival among males and Panel (**D**) depicts differences among females of SP-A1 (6A^2^, 6A^4^), SP-A2 (1A^0^, 1A^3^), and KO. Significant differences in survival are indicated * *p* < 0.05 (log-rank test and one-way analysis of variance (ANOVA) followed by Bonferroni multiple comparisons correction). The number of mice used for each group is shown in Figure 2, ns: not significant.

SP-A1: Bacterial infection resulted in significant differences in survival between males and females of the 6A^2^ variant only (Figure 3A) where males showed a significantly lower survival compared, not only to 6A^2^ females but also to 6A^4^ males and females.

SP-A2: The 1A^0^ and 1A^3^ males and females exhibited no significant sex differences in survival (Figure 3B). However, the 1A^0^ females had significantly higher survival compared to 1A^3^ males and females (Figure 3B).

#### 3.2.2. Differences among SP-A1 and SP-A2 Variants

The SP-A2 (1A^0^) males exhibited a significantly higher survival compared to SP-A1 (6A^2^) or KO males. The survival of 1A^3^ and 6A^4^ males, although lower than the 1A^0^, was not significantly different (Figure 3C). The survival of 1A^3^ males was not significantly different than 6A^2^ males (Figure 3C). The 1A^0^ females, although they showed a trend of higher survival, this was not significantly different from 1A^3^, 6A^2^, 6A^4^, and KO females (Figure 3D), whereas the 1A^3^ females had similar survival compared to 6A^2^, 6A^4^ and KO females (Figure 3D).

### 3.3. Effect of SP-A Variants on Survival in Response to O_3_ or FA Exposure Prior to Infection

**Figure 4:**
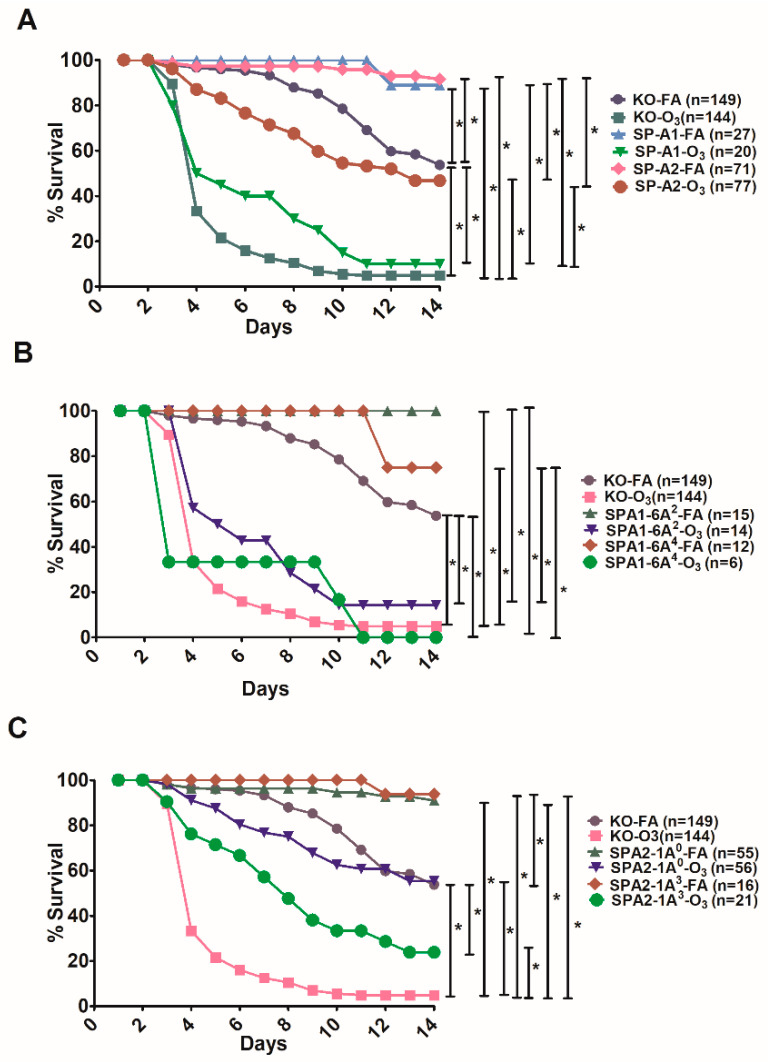
Gene- and variant-specific survival of mice exposed to FA or O_3_ prior to *K. pneumoniae* infection. Panel (**A**) depicts differences in daily survival of male and female mice carrying a single SP-A1 or SP-A2 variant and those lacking SP-A (KO). Panel (**B**) depicts differences in daily survival of KO and each SP-A1 (6A^2^, 6A^4^) variant, and Panel (**C**) depicts differences in survival of KO and each SP-A2 (1A^0^, 1A^3^) variant. In all panels, males and females are combined after FA or O_3_ exposure and infection. Significant differences are indicated for survival * *p* < 0.05 (log-rank test and one-way analysis of variance (ANOVA) followed by Bonferroni multiple comparisons correction).

All mice had a significantly decreased survival after O_3_ exposure and infection, compared to FA-exposed and infected mice (Figure 4A). The survival of SP-A2 mice (male and female combined) exposed to O_3_ and infection was significantly better compared to KO or SP-A1 (Figure 4A). Moreover, the survival of SP-A-KO FA-exposed animals was similar to SP-A2 ozone-exposed animals and significantly lower than that of SP-A1 and SP-A2 FA-exposed animals (Figure 4A).

When the two SP-A1 and SP-A2 variants were analyzed separately, a similar observation was made for the SP-A1 variants as when they were analyzed together (Figure 4B). Both 6A^2^ and 6A^4^, as well as KO, showed a similar survival in response to O_3_ and this was significantly reduced compared to their corresponding FA-exposed groups. The SP-A2 variants, on the other hand, showed significant differences in response to O_3_ exposure and infection with the 1A^3^ showing a significantly lower survival compared to 1A^0^, but had significantly higher survival than KO (Figure 4C). The 1A^0^ exhibited similar survival as the SP-A-KO FA-exposed animals (Figure 4C), as shown with the combined SP-A2 variants in Figure 4A.

### 3.4. Differences in Survival as A Function of Variant in Male and Female SP-A2 and KO-Infected Mice with Prior FA or O_3_ Exposure

**Figure 5:**
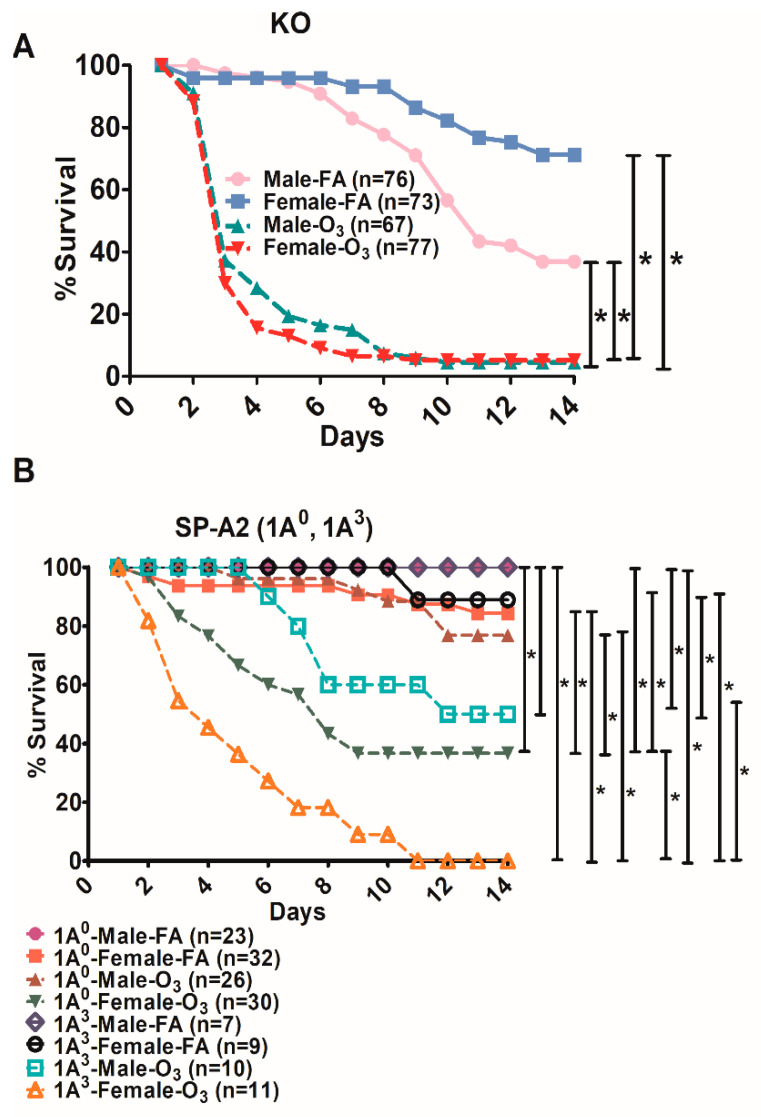
Effect of prior FA or O_3_ exposure on survival of mice after *K. pneumoniae* infection as a function of sex. The survival rate was measured in SP-A-KO (panel (**A**)) and SP-A2 (1A^0^, 1A^3^) (panel (**B**)) male and female mice over a period of 14 days after FA or O_3_ exposure and infection. Although the number for the SP-A1 mice (not shown) was very small, a trend similar to that for KO was observed for each SP-A1 variant. Significant differences for survival are indicated * *p* < 0.05 (log-rank test and one-way analysis of variance (ANOVA) followed by Bonferroni multiple comparisons correction). The number of mice used in each group is shown in Figure panels in parenthesis (*n* =). FA exposed shown in solid line and O_3_ exposed shown in broken line.

O_3_ exposure and infection with *K. pneumoniae* resulted in significant differences in survival over time. In the SP-A2 (1A^3^) and KO groups, all males and females showed a significant decrease in survival after O_3_ exposure and infection compared to FA and infection (Figure 5A,B). In contrast, the SP-A2 (1A^0^) males did not show any significant differences between FA- and O_3_-exposed animals, although significant differences were observed between 1A^0^ males and females in response to O_3_ exposure. For the SP-A1 (6A^2^, 6A^4^) groups, the numbers were too small once each group was divided into male and female subgroups; however, the trend was similar to that observed for KO. No significant changes were observed among the FA-exposed groups.

### 3.5. Comparison of Survival between Animals with Infection Alone and Animals with FA Exposure Prior to Infection

**Figure 6:**
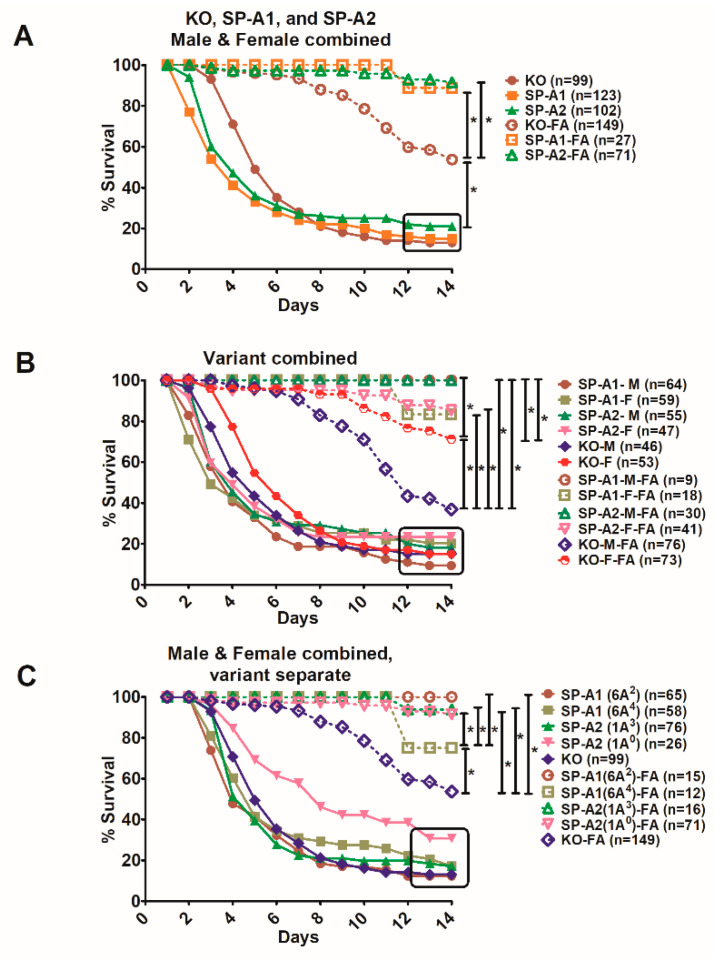
Comparison of survival after *K. pneumoniae* infection alone and infected mice with prior FA exposure. Differences in daily survival rate of male and female mice was measured after infection or FA exposure prior to infection. Panel (**A**) depicts the survival curves of SP-A1, SP-A2, and KO-infected mice, and those of FA-infected mice. Panel (**B**) depicts survival curves for the same groups as in Panel A, but as a function of sex. Panel (**C**) depicts survival curves as a function of SP-A1 and SP-A2 variants. In all panels, the dotted lines with open marks depict the FA-infection and are significantly different (*p* < 0.05, log-rank test and one-way analysis of variance (ANOVA) followed by Bonferroni multiple comparisons correction) from infection alone (closed marks, all curves are enclosed at the 12–14-day survival by a black square). All other significant differences (*p* < 0.05, log-rank test and one-way analysis of variance (ANOVA) followed by Bonferroni multiple comparisons correction) are indicated by bars. The number of mice used in each group is shown in Figure panels in parenthesis (*n* =).

The FA exposure prior to infection was initially used to serve as a control for the ozone-exposed animals prior to infection. We expected that the survival of animals with FA exposure prior to infection would be similar to that of infected animals without prior FA exposure. However, to our surprise, this was not the case. The survival curves of the studied groups under these two conditions are shown in Figure 6.

The exposure of mice to FA prior to *K. pneumoniae* infection resulted unexpectedly in a major and significant increase in survival over time compared to those that were not exposed to FA prior to infection. The SP-A1 (6A^2^, 6A^4^), SP-A2 (1A^0^, 1A^3^) variants, as well as KO males and females exhibited better survival compared to the corresponding mouse groups infected with *K. pneumoniae* alone (Figure 6A). The KO FA-exposed animals showed a significant decrease in survival compared to FA-exposed SP-A1 and SP-A2, indicating a positive impact of SP-A on survival. An overall similar observation was made when SP-A1 and SP-A2 males and females were analyzed separately (Figure 6B). The infected alone showed poor survival and were significantly different from those exposed to FA prior to infection. FA-exposed KO males showed a significantly lower survival compared to FA-exposed KO females and FA-exposed SP-A1 and SP-A2 males and females. The FA-exposed SP-A1 and SP-A2 males exhibited a better survival than the corresponding female groups without reaching significant differences (Figure 6B). When survival was studied for each SP-A1 and SP-A2 variant with males and females combined, a similar observation was made overall (Figure 6C). All variants and KO-infected mice showed a lower survival rate compared to mice infected with prior FA exposure. The FA-infected 6A^4^ mice had a significantly lower survival (*p* < 0.05) than all other FA-infected 1A^0^, 1A^3^, and 6A^2^ mice (Figure 6C). A summary of the cumulative survival for all the groups studied under the different conditions is shown in Table 1.

**Figure 7:**
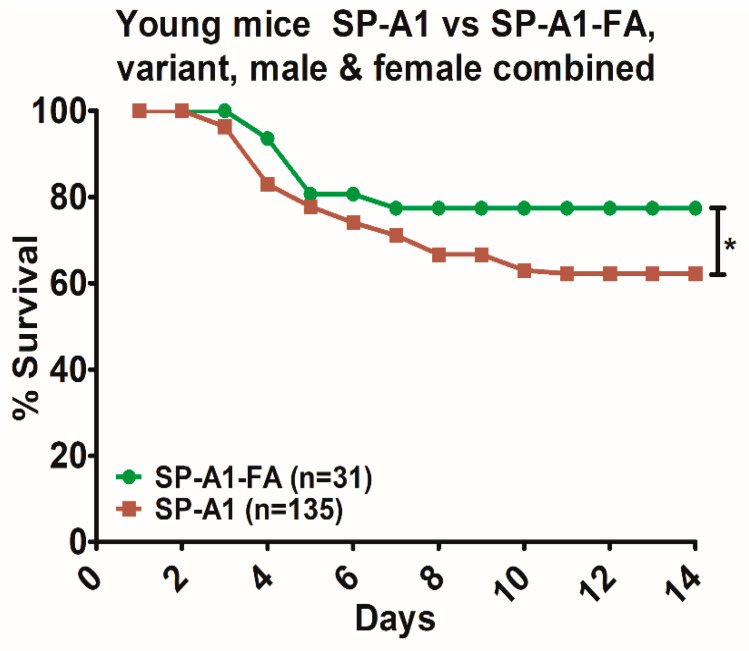
Comparison of survival in young mice after *K. pneumoniae* infection alone and infected mice with prior FA exposure. Differences in daily survival rate of young SP-A1 male and female mice was measured after infection, published data [39], or FA exposure prior to infection (present data). Significant differences in survival are indicated * *p* < 0.05 (Chi-Square and log-rank test). The number of mice used in each group is shown in Figure panels in parenthesis (*n* =).

Next, in a pilot study using young mice (~3–4 months), we compared the survival of SP-A1 mice exposed to FA prior to infection to that of our published data of SP-A1 infected alone [39], in order to determine whether this improved survival with prior FA exposure observed in aged mice was also occurring in young mice. A significant increase in survival over time was observed for SP-A1-FA-exposed compared to those that were not exposed to FA prior to infection (Figure 7). However, the magnitude of change was considerably lower than that observed for the aged mice (compare Figure 6A with Figure 7).

## 4. Discussion

Surfactant protein A (SP-A) plays an important role in lung innate immunity. The surfactant protein A1 (SP-A1) and SP-A2 exhibit differences in their ability to enhance phagocytosis by AM, and SP-A2 is more effective than SP-A1 [34,35,83]. The lung microenvironment affects the functional activity of a given variant. For example, an increase in reactive oxygen species (ROS) differentially oxidizes SP-A variants, and this consequently has an effect on their function. Oxidative stress is shown to affect SP-A2 gene-specific variants activity more than SP-A1 variants, although the oxidized SP-A2 molecules are still more active than the oxidized SP-A1 variants [84]. Previous studies have shown that the SP-A1 and SP-A2 variants differentially affect airway mechanics [40] and survival [39] in young mice in response to *Klebsiella pneumoniae* infection. The SP-A2 (1A^0^, 1A^3^) variants exhibited better survival than the SP-A1 variants [39] in response to infection. In the current study, we investigated whether SP-A variants differentially affect survival of aged mice (~9–12 months) in response to infection, as well as in response to infection and oxidative stress. For this purpose, we either infected animals or exposed the animals to ozone (O_3_) or filtered air (FA, control) prior to infection, and then studied their daily survival over a 14-day period. This study included hTG mice carrying a different SP-A1 or SP-A2 variant as well as mice lacking SP-A (i.e., KO). **In response to infection alone,** we observed (a) no gene-specific survival SP-A1 = SP-A2 = KO (male and female combined); (b) variant-specific survival SP-A2 (1A^0^) > 1A^3^ = 6A^2^ = 6A^4^ (male and female combined); (c) Sex differences in survival in SP-A1 (6A^2^) mice, with females showing a better survival that males; but no sex difference in SP-A2 (1A^0^, 1A^3^), SP-A1 (6A^4^), and KO mice. **In response to O_3_ or FA exposure and subsequent infection,** we observed (d) gene-specific survival SP-A2 > SP-A1 = KO (male and female combined) in O_3_-exposed infected animals, but no gene-specific differences in FA-exposed infected animals (SP-A2 = SP-A1 > KO); (e) variant-specific survival SP-A2 (1A^0^) > 1A^3^ = 6A^2^ = 6A^4^ (male and female combined) in O_3_-exposed but not in FA-exposed mice; (f) male and female SP-A1, SP-A2, and KO O_3_-exposed infected mice exhibited lower survival compared to control (infected and filtered air (FA) exposed) mice, except for the SP-A2 (1A^0^) males that in response to O_3_ exhibited better survival compared to females and the 1A^0^ male survival was similar to that of FA-exposed males and females. **Comparison of infected mice with and without prior FA exposure** resulted in an unexpected finding. Compared to the survival of infected mice alone, a significantly better survival was observed in males and females of FA-exposed mice prior to infection in all mouse strains (SP-A1, SP-A2, SP-A-KO) in both aged and young mice. However, the magnitude was considerably larger in aged mice. This serendipitous finding, as discussed below, may have clinical relevance especially in the current pandemic. Collectively, these observations indicate: (a) that the SP-A2 (1A^0^) variant is more effective in combating the harmful effects of various environmental insults, such as infection and oxidative stress, especially in male mice, and (b) that increased ventilation (i.e., with a high airflow rate), as provided here by FA exposure, prior to infection has a significantly positive impact on survival.

AM reside in the hypophase, i.e., the liquid between the air and lung alveolar cells, and provide the first-line of defense against invading harmful pathogens via phagocytosis and killing of invading pathogens and by modulating the innate immune response [85]. However, when the number of invading pathogens is overwhelming or pathogens are too virulent and are uncontrollable by AM alone, AM produce chemokines and other inflammatory mediators and recruit neutrophils from the pulmonary vasculature into the alveolar space to enhance the host defense [86]. In addition to the cell recruitment, the phagocytes produce reactive oxygen and nitrogen intermediates that are important for the clearance of bacteria [87]. SP-A is involved in multiple AM-mediated host defense functions such as the stimulation of chemotaxis of macrophages [17] and enhancement of phagocytosis of bacteria by macrophages [18,19]. Previous studies have shown SP-A enhances phagocytosis of klebsiella by two mechanisms, one of which is by serving as an opsonin, which binds to the capsular polysaccharides of the bacteria and potentially to SP-A receptors on the macrophages, and the other by activating the macrophages, resulting in increased activity of the mannose receptor [88]. Our previous studies have shown that, the SP-A variants, SP-A1 and SP-A2, exhibit differences in their ability to enhance phagocytosis by AM, and SP-A2 is more effective than SP-A1 [34,35].

### 4.1. Young vs Aged

The comparison of cumulative survival of aged mice with our published young mice [39] (Table 1) in response to *K. pneumoniae* infection, showed a significant decrease in the survival of aged male and female mice expressing SP-A variants as well as in KO mice compared to previously published young mouse survival [39]. A number of factors may contribute to this. Mouse AM are shown to express reduced levels of heme oxygenase-1 with aging, [89] and this may have a negative effect on the protection against oxidative stress in old age. Studies of humans and rodents (primarily rats) have been extensively used to study aging and have shown that the relative proportion of AM in bronchoalveolar lavage is reduced as a function of age [90,91]. AM from old rats displayed reduced production of TNF-α and IL-10 [64,65]. However, it was found that the phagocytic ability of rat AM is increased with aging in response to *K. pneumoniae* infection [92], but the same study also showed that the lung-recruited neutrophils exhibited reduced phagocytosis of *K. pneumoniae* [92]. Moreover, the expression levels of MAP kinases ERK, p38, and JNK were reduced in aged mice compared to younger mice [93,94,95]. AM from younger rats expressed p56 and p54 isoforms of JNK, whereas older rats expressed p54 and p46 isoforms [96], indicating that the expression levels and isoforms of molecules involved in MAP kinases differ in aged animals. Furthermore, the activation of macrophages triggers signaling networks that are found to be altered in aged individuals. These may include, a decreased expression [95] or activation [96] of the NF-kB pathway, a reduction in the TLR-signaling pathway that leads to NF-kB activation, and the adaptor molecules, MyD88, as well as members of the NF-κB pathway (Rel-a, Rel-b, NF-κB p50, and p52, and TRAF6) [95]. Collectively, these data indicate that the reduced phagocytosis by the recruited neutrophils, the reduced number of AM, a reduction in heme oxygenase-1 level, and an impairment in the production of both pro-inflammatory and anti-inflammatory cytokines, as well as a compromise in signaling pathways, may contribute to a defective immunosurveillance resulting in an increased susceptibility to respiratory infection in old age.

Of note, the aged mice, when exposed in pilot experiments to *K. pneumoniae* inoculum at the same dose as young mice, exhibited a successful innate immune response, and with or without the need of support of acquired immunity, were able to eliminate the pathogen (all mice survived). However, when the dose of *K. pneumoniae* inoculum was four times the dose used in young mice, the balanced immune response got dysregulated, and a dramatic shift towards an uncontrolled pro-inflammatory state followed resulting in higher mortality compared to young infected mice. As discussed above, the age-related differences in immune responses to bacterial infections are complex, in rodents and in humans. Our survival data of infected aged mice reveal that exposure to an overwhelming bacterial inoculum is required for the immune responses to fail and for rapid disease progression to follow. The need, compared to young mice (450 CFU/µL, for a higher dose of *K. pneumoniae* inoculum (1800 CFU/5 µL)), in order to develop an aged mouse survival model, indicates an important role for the mature immune system in resistance to disease in infected aged mice. While the “less” mature immune system of the young mice may make them vulnerable to a lower dose of the *K. pneumoniae* inoculum, the probable development of uncontrolled local and systemic inflammation occurs less frequently at this dose (as reflected in the lower mortality rate) and possibly requires the presence of other host variables. Also, a better baseline lung function in young mice may contribute to recovery after exposure to this lower dose of the bacterial inoculum. These factors may account for the higher level of survival of young mice compared to aged mice after exposure to infection alone. Thus, it appears that the susceptibility of young mice to *K. pneumoniae* inoculum is derived from a “less” mature immune system, whereas, the susceptibility of aged mice is mediated by uncontrolled inflammation triggered by a large dose of *K. pneumoniae*.

### 4.2. In Response to Infection Alone

The overall survival rate of aged mice in all groups was significantly lower than that previously observed in young mice [39]. Aging is associated with a decrease in the function of innate immune cells, macrophages, neutrophils, and dendritic cells (DC), and this results in an unexpected increase in inflammatory response with age [97].

Young mice exhibited SP-A variant- and sex-specific differences in survival after infection. The SP-A2 (1A^0^) variant was associated with high survival in both males and females, although females exhibited a small, but significantly better survival than males [39]. In humans, the SP-A2 (1A^0^) was found to associate with better survival in lung transplant patients who tend to be older in age. This better survival was observed especially in the first year after lung transplant; this is the most critical time perhaps due to dysregulation of inflammation and host defense [98]. In the present study, SP-A2 (1A^0^) in aged mice, although it didn’t show any sex differences, was associated with a significantly better survival when both male and female mice were combined (Figure 1B) (Table 1), as observed in lung transplant patients [98]. These together indicate that the better survival observed in the presence of 1A^0^ is independent of age, and the small sex differences observed in young mice [39] are eliminated in aged mice (current study) and in older humans [98]. The SP-A1 (6A^2^) mice, on the other hand, exhibited sex differences with females showing a better survival than males (Table 1; Figure 2B), whereas none of the other variants exhibited sex differences in aged mice. The latter is in contrast to sex differences observed for all SP-A1 and SP-A2 variants studied in the young mouse survival studies, indicating that sex differences are eliminated with old age for all but one of the SP-A variants.

The SP-A1 (6A^2^) mice compared to SP-A2 (1A^0^) exhibited a significantly reduced survival in males but not in females, and although a trend toward a higher rate of survival was observed in 1A^0^ females, this didn’t reach statistical significance. The SP-A1 and SP-A2 genes, and their corresponding variants, differ in the coding region [28,31] by four amino acids at residues 66, 73, 81, and 85 in the collagen-like domain. The amino acid at position 85 of the precursor molecule, where SP-A1 has a cysteine and SP-A2 has an arginine [28], has a major impact on SP-A oligomerization, lipopolysaccharide (LPS) aggregation, and phagocytosis [42]. Moreover, the presence of cysteine in the collage-like domain in SP-A1 may cause a micro-instability resulting in a less stable protein [32]. It is possible that this micro-instability may modulate functions mediated by the carbohydrate recognition domain (CRD) region that binds, among others, bacteria and allergens [11,12,99,100]. In addition, apart from the gene-specific amino acid differences, the SP-A2 (1A^0^) differs from the SP-A1 (6A^2^) at residues 19 and 91. The former may or may not be part of the signal peptide [41] and the latter is part of the collagen-like domain [101] holding the potential to contribute to molecular stability. Collectively, the gene-specific and variant-specific amino acid differences may contribute to overall functional differences of 6A^2^ and 1A^0^, but currently, the underlying mechanisms are not known.

### 4.3. In Response to O_3_ or FA and Infection

Both lung function and innate host defense are significantly affected by air pollutants, such as ozone (O_3_) [66]. O_3_ exposure is shown to significantly affect females more than males in several lung diseases [102,103,104,105]. Significant sex differences in survival after infection and O_3_ exposure have been observed with females being more susceptible to oxidative stress than males [80,106]. Sex hormones have been shown to play a role in sex-dependent survival [107]. Moreover, SP-A has been shown to have a positive impact on survival compared to the SP-A-KO mice [24]. In the present study, the aged SP-A1 (6A^2^, 6A^4^) and KO mice exhibited a similar and a significantly decreased survival in response to O_3_ exposure and infection compared to SP-A2 (1A^0^, 1A^3^) mice. Interestingly, the survival rate of the FA-exposed SP-A-KO mice was similar to that of O_3_-exposed SP-A2 mice, indicating that host defense deficits in the KO may have contributed to its low rate of survival even in the absence of O_3_ exposure. A finding consistent with these observations has been observed previously in a proteomics study of the alveolar macrophages where the protein levels in FA-exposed KO mice were closer to ozone-exposed wild type mice, indicating that the SP-A-KO mice may be in a state of chronic oxidative stress [108].

Of the two SP-A2 variants, the 1A^0^ compared to 1A^3^ exhibited significantly better survival in females. Males (1A^0^), on the other hand, exhibited the highest survival among the studied groups after O_3_, and although this differed significantly from that observed in 1A^0^ females, it did not differ significantly from the 1A^3^ males or FA-exposed males and females. These sex- and variant-dependent differences point to the complexities of the interplay of genetics and sex. In this particular case, the SP-A2 variants differ only at a single amino acid at residue 223 (Gln for 1A^0^ and Lys for 1A^3^) [28], both of which are hydrophilic amino acids with polar charged (Gln) or uncharged (Lys) side chains. Residue 223 is located within the CRD and the findings underscore the importance of the CRD in survival in response to ozone-induced oxidative stress and infection. It also points to the possibility that this single amino acid, either by itself or within its surrounding amino acid context, mediates, directly or indirectly, sex-specific pathways.

It has been shown that O_3_ exposure has a negative impact on the phagocytic activity of macrophages, and AM from female mice exhibited lower activity than male mice after O_3_ exposure [80]. Moreover, the phagocytic activity of SP-A2 (1A^0^) is higher than the SP-A1 variants [34], even though its phagocytic activity is affected more than SP-A1 in response to O_3_ exposure [84]. Differences in the phagocytic index between SP-A2 variants (1A^0^, 1A^1^) have been observed in response to *Pseudomonas aeruginosa* infection, where the 1A^1^ variant exhibited a higher phagocytic index than the 1A^0^ [35]. The 1A^1^ variant differs from the 1A^3^ (discussed above) only at amino acid 9 within the signal peptide. Thus, the mature proteins (minus the signal peptide) of 1A^1^ and 1A^3^ are identical in the amino acid sequence that includes of course residue 223. The observed functional difference between 1A^1^ and 1A^0^ provides further support of the importance of residue 223 in the SP-A function. Further studies are needed to study functional differences between these variants in response to oxidative stress. Based on these observations, we speculate that aged males carrying the 1A^0^ and perhaps the 1A^3^ genetic variant may exhibit a genetic survival advantage over females and those carrying a different SP-A2 or SP-A1 variant in response to ozone pollution and infection. Furthermore, a differential functional impairment of host defense molecules, such as the SP-A variants, as it may occur in response to oxidative stress may, in part, explain differences in clinical outcomes, and be one of the mechanisms that contributes to the increased risk of hospitalization for pneumonia [109,110,111].

### 4.4. In Response to Infection with Prior Filtered Air Exposure

The unexpected and surprising finding that mice with prior FA exposure exhibit significantly better survival compared to mice with infection alone (with no prior FA exposure) was difficult at first to understand and explain. After further evaluation of the exposure system, and guided by the available literature and expert input, this finding may not be as surprising as originally thought. The system we use, which was set up about 20 years ago, delivers an airflow of 15 L/min to the exposure vessel where mice are placed during the exposure. A flow rate of 15 L/min was considered reasonable for a small exposure vessel to ensure no buildup of CO_2_ (from the animals) during the course of an experiment, reduce odor and ammonia build-up in the chamber, and provide an overall adequate number of air exchanges/h. However, in exposure system settings from other labs with smaller exposure vessels, the airflow was set to allow 30 air volume exchanges/h [112]. In our system [23,82], with an exposure vessel size of 3.66 L, the flow rate we used resulted in four complete air changes/minute, which is considerably higher than that used earlier in other similar systems. Also of note is that the FA is humidified and warmed to a temperature of 37.5 °C, prior to delivery into the exposure vessel, and the exposure vessel is maintained at a constant positive pressure of 1.5 cm H_2_O relative to the surroundings, to protect against contamination in the event of a leak and as a method for detection of leaks [82,113,114].

With this information in mind, we postulate that the 3 h of exposure to high flow FA (i.e., even though it does not have increased fraction of inspired O_2_) would improve overall ventilation of the mouse lung. This effect is mediated by the development of positive nasopharyngeal and tracheal airway pressures from the effect of exposure to warm, humidified air, and the Positive End Expiratory Pressure (PEEP)-like effect of the positive pressure in the exposure vessel combined with the high flow rate [115,116,117,118,119,120,121]. This increased lung ventilation is associated with greater alveolar recruitment and end-expiratory lung volumes, better alveolar gas exchange, and thus improves the Ventilation/Perfusion (V/Q) match and decreases the work of breathing [115,122,123,124]. Following 3 h of exposure to high flow FA, the mice are expected to be in an improved pulmonary function state and better oxygenated with more of the lung reserve having been recruited. Therefore, it is conceivable that the oropharyngeal inoculation/aspiration of *K. pneumoniae* will have a differential effect on the two groups of mice, i.e., the infected mice without prior FA exposure and the infected mice with prior FA exposure.

These differences may range from the early host defense mechanisms of the lung to the development and severity of the pneumonia, and whether respiratory failure and sepsis develop. The greater alveolar recruitment in the high flow FA-exposed mice would result in the same amount of *K. pneumoniae* inoculum to be spread over a larger surface area of the lung. We propose that this relative decrease in the bacterial density of distribution may allow for a greater efficacy of the pulmonary host defense mechanisms, such as the muco-ciliary trapping and elimination of the bacteria, the innate immune functions of molecules such as SP-A and of the alveolar macrophages, and the transition from innate immunity to acquired immunity. The effectiveness of these phases of host response is critical in determining whether the local and systemic inflammatory responses will limit the local multiplication of bacteria and the degree of damage to the alveoli or whether the more rapid multiplication of the bacteria will lead to greater alveolar damage and uncontrolled local and systemic inflammation, and subsequent complications of respiratory failure and sepsis [87,125,126,127]. Atelectasis is considered to be a risk factor for lung infections and opening up collapsed alveolar spaces and recruiting alveoli is thought to reduce the risk, as exemplified by the postoperative use of Incentive Spirometry, alone or as a part of the I COUGH program, and by the prophylactic use of nasal Continuous Positive Airway Pressure postoperatively in patients undergoing cardiac surgery [128,129]. In summary, this serendipitous finding indicates that good lung ventilation could mitigate the deleterious effects of a Gram-negative infection, and most importantly, improve survival, as shown here in aged mice and to a lesser degree in young mice, where in the absence of increased ventilation, considerably lower survival rates occurred. We postulate that this increased ventilation works synergistically with innate immunity as provided by molecules such as the SP-A variants and the alveolar macrophages [130], and lead to a successful first line of defense that minimizes/eliminates dire downstream consequences. Thus, our serendipitous finding has relevance to clinical settings. There may be a need to explore whether the periodic use of High Flow Nasal Cannula (HFNC) set at Fraction of Inspired Oxygen (FiO_2_) of 0.21 or higher, if needed, in patients at high risk for the development of pulmonary infections, would lead to lower infection rates and better outcomes. In view of the current, ongoing SARS-CoV-2 pandemic, where aged individuals are at a higher risk of severe COVID-19 disease and mortality, examining the role of prophylactic HFNC would be valuable.

## 5. Conclusions

In conclusion: (a) the gene-/variant- and sex-specific survival with a few exceptions are largely eliminated in aged mice in response to infection. The SP-A1 (6A^2^) showed better survival in females than males and the SP-A2 (1A^0^) variant exhibited better survival in response to infection, especially in males. The 1A^0^ also showed a better survival in response to O_3_ in both males and females. SP-A variants have an overall positive impact on survival compared to SP-A-KO mice in response to infection with prior FA exposure in both males and females. (b) Better ventilation as a result of prior FA exposure results in a significantly better survival. Although both young and aged mice enjoyed the survival benefit with prior FA exposure, the magnitude of the survival benefit was larger in the aged mice. Clinical studies are needed to explore whether HFNC has the potential to provide comparable benefits in humans. It is a fairly simple and minimally invasive approach that if applied in the clinic and works, as shown here with mice, could save precious lives, especially in the aged population.

## Figures and Tables

**Table 1:** Cumulative survival (%) of aged SP-A1, SP-A2, and KO males and females separately as well as combined in response (a) to *K. pneumoniae* infection alone, and (b) after FA or O_3_ exposure followed by infection.

Variants and Sex		^†^ Young Infection (~450 CFU)	Aged Infection (~1800 CFU)	Aged-FA + Infection (~1800 CFU)	Aged-O_3_ + Infection (~1800 CFU)	O_3_/FA	* *p*-Value
**KO**	**Female**	47.16	15.09	71.23	5.19	7.29	<0.05
	**Male**	36.17	10.86	36.84	4.47	10.71	
**6A^2^**	**Female**	79.06	21.21	100	14.28	11.11	
	**Male**	48.64	3.12	100	14.28	16.66	
**6A^4^**	**Female**	66.66	19.23	66.66	0	0	
	**Male**	42.85	15.62	100	0	0	
**1A^0^**	**Female**	96.77	36.36	84.37	36.66	40.74	
	**Male**	83.87	26.66	100	76.92	86.95	
**1A^3^**	**Female**	85.71	19.44	88.88	0	0	
	**Male**	46.87	15	100	50	71.42	
**Male and Female combined**							
**Variants**		**^†^ Young mice (~450 CFU)**	**Aged infection (~1800 CFU)**	**Aged - FA + infection (~1800 CFU)**	**Aged - O_3_ + infection (~1800 CFU)**	**O_3_/FA**	* *p*-Value
**KO**		42	13.13	53.69	4.86	8.75	<0.05
**6A^2^**		65	12.3	100	14.28	13.33	
**6A^4^**		54.54	17.24	75	0	0	
**1A^0^**		90.32	30.76	90.9	55.35	62	
**1A^3^**		68.91	17.1	93.75	23.8	33.33	

* Significant differences were observed in survival *p* < 0.05 for the following comparisons: (i) young vs. aged infected mice, (ii) aged infection vs. aged -FA-exposed + infection, and (iii) aged -FA-exposed + infection vs. aged O_3_-exposed + infection mice. ^†^ Published survival study data [39], were utilized to compare the cumulative survival between young and aged mice. SP-A2 (1A^0^), which is the one or one of the variants associated with better survival in all conditions studied, is highlighted in yellow. O_3_/FA: indicates the ratio of difference in percentage of mice survived in response to O_3_ compared to FA.
